# Supplementary material for: The SAM domain of mouse SAMHD1 is critical for its activation and regulation
Source: Nat Commun. 2018 Jan 29;9:411. doi: 10.1038/s41467-017-02783-8 (PMC5788916; doi:10.1038/s41467-017-02783-8)
Supplement: Supplementary file 1 — Supplementary Information [file 41467_2017_2783_MOESM1_ESM.pdf]

Supplementary Figure 1

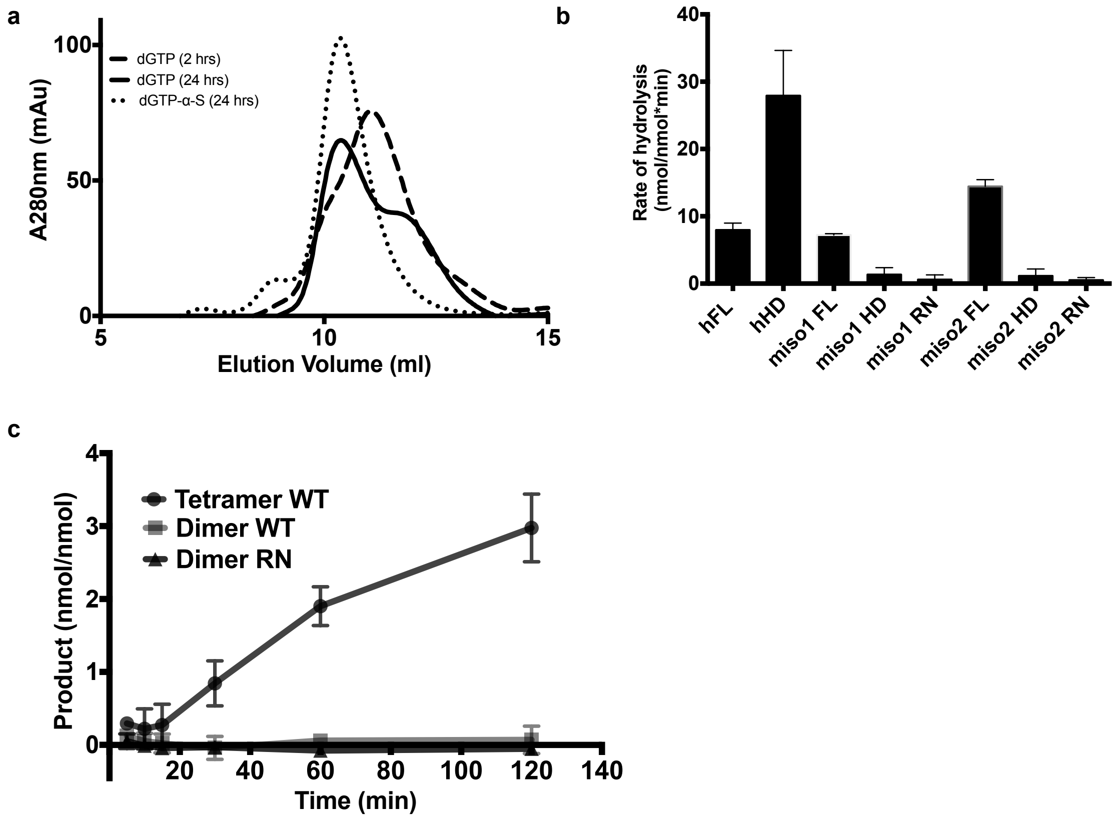

Supplementary Figure 1. mSAMHD1 tetramerization and activity. (**a**) SEC analysis of purified full-length mSAMHD1 iso1 RN mutant incubated with dGTP or dGTP-α-S for 2 or 24 hours. (**b**) dNTPase assay for full-length (FL), HD, and RN constructs of hSAMHD1 and mSAMHD1. Reaction products were quantified by the malachite green assay. Each experiment was performed in triplicate. Error bars, s.d. (**c**) dNTPase activity assay for mSAMHD1 iso2 dimer or tetramer formed during purification, without additional allosteric nucleotides added to the reaction mixtures. Tetramers formed in the absence of additional allosteric nucleotides have very low activity requiring incubation times of 2 hours. Reaction products were quantified by the malachite green assay. Each experiment was performed in triplicate. Error bars, s.d.

Supplementary Figure 2

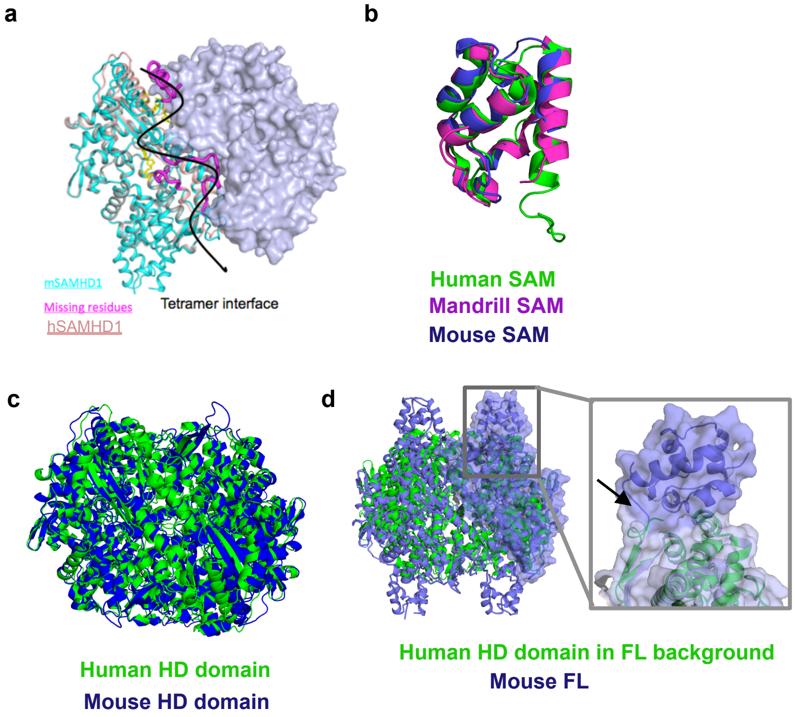

Supplementary Figure 2. mSAMHD1 is structurally similar to hSAMHD1. (**a**) Structural alignment of the mSAMHD1 structure (cyan and purple surface) to the hSAMHD1 structure PDB ID 4BZB (pink), with unobserved mSAMHD1 residues highlighted with magenta tubes. (**b**) Structural alignment of the SAM domain from the current full-length mSAMHD1 structure (blue), the crystal structure of human SAM domain (PDBID 2E8O) (green), and the NMR structure of the mandrill SAM domain (PDBID 5AJA) (magenta). (**c**) Structural alignment of the HD domains from the current full-length mSAMHD1 structure and the full-length hSAMHD1 structure (PDBID 5AO0) (green) in which the SAM was not observed. (**d**) Structural alignment of the full-length hSAMHD1 structure (SAM unobserved) (green) (PDBID 5AO0) and the current full-length mSAMHD1 structure (purple). Arrow indicates the largest difference between the structures at the HD-SAM interface.

Supplementary Figure 3

a

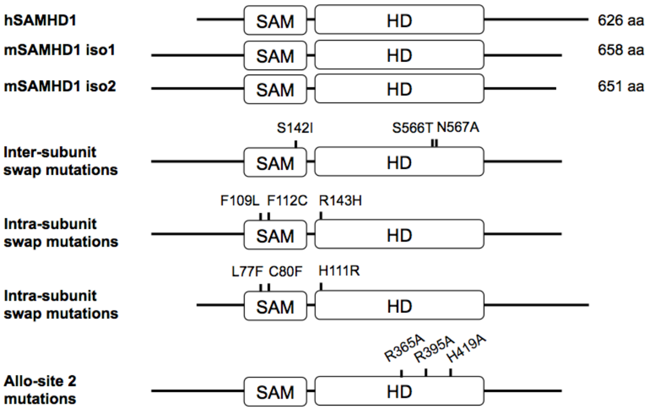

b

```
                    1        10        20        30        40        50        60
mSAMHD1_isoform1    MDSLLGCGVSAAAREPVPRYLTSQPRVSEVAMQSAPLEQPAKRPRCDGSPRTPPSTPPAT
mSAMHD1_isoform2    MDSLLGCGVSAAAREPVPRYLTSQPRVSEVAMQSAPLEQPAKRPRCDGSPRTPPSTPPAT
hSAMHD1             ...............................MQRADSEQPSKRPRCDDSPRTPSNTPSAE

                             70        80        90       100       110       120
mSAMHD1_isoform1    ANLSADDDFQNTDLRTWEPEDVCSFLENRGFREKKVLDIFRDNKIAGSFLPFLDEDRLED
mSAMHD1_isoform2    ANLSADDDFQNTDLRTWEPEDVCSFLENRGFREKKVLDIFRDNKIAGSFLPFLDEDRLED
hSAMHD1             ADWSPGLEL.HPDYKTWGPEQVCSFLRRGGFEEPVLLKNIRENEITGALLPCLDESRFEN

                            130       140       150       160       170       180
mSAMHD1_isoform1    LGVSSLEERKKMIECIQQLSQSRIDLMKVFNDPIHGHIEFHPLLIRIIDTPQFQRLRYIK
mSAMHD1_isoform2    LGVSSLEERKKMIECIQQLSQSRIDLMKVFNDPIHGHIEFHPLLIRIIDTPQFQRLRYIK
hSAMHD1             LGVSSLGERKKLLSYIQRLVQIHVDTMKVINDPIHGHIELHPLLVRIIDTPQFQRLRYIK

                            190       200       210       220       230       240
mSAMHD1_isoform1    QLGGGYYVFPGASHNRFEHSLGVGYLAGCLVRALAEKQPELQISERDILCVQIAGLCHDL
mSAMHD1_isoform2    QLGGGYYVFPGASHNRFEHSLGVGYLAGCLVRALAEKQPELQISERDILCVQIAGLCHDL
hSAMHD1             QLGGGYYVFPGASHNRFEHSLGVGYLAGCLVHALGEKQPELQISERDVLCVQIAGLCHDL

                            250       260       270       280       290       300
mSAMHD1_isoform1    GHGPFSHMFDGRFIPRARPEKKWKHEQGSIEMFEHLVNSNELKLVMKNYGLVPEEDITFI
mSAMHD1_isoform2    GHGPFSHMFDGRFIPRARPEKKWKHEQGSIEMFEHLVNSNELKLVMKNYGLVPEEDITFI
hSAMHD1             GHGPFSHMFDGRFIPLARPEVKWTHEQGSVMMFEHLINSNGIKPVMEQYGLIPEEDICFI

                            310       320       330       340       350       360
mSAMHD1_isoform1    KEQIMGPPITPVKDSLWPYKGRPATKSFLYEIVSNKRNGIDVDKWDYFARDCHHLGIQNN
mSAMHD1_isoform2    KEQIMGPPITPVKDSLWPYKGRPATKSFLYEIVSNKRNGIDVDKWDYFARDCHHLGIQNN
hSAMHD1             KEQIVGPLESPVEDSLWPYKGRPENKSFLYEIVSNKRNGIDVDKWDYFARDCHHLGIQNN

                            370       380       390       400       410       420
mSAMHD1_isoform1    FDYKRFIKFARICEVEYKVKEDKTYIRKVKHICSREKEVGNLYDMFHTRNCLHRRAYQHK
mSAMHD1_isoform2    FDYKRFIKFARICEVEYKVKEDKTYIRKVKHICSREKEVGNLYDMFHTRNCLHRRAYQHK
hSAMHD1             FDYKRFIKFARVCEVDNE...........LRICARDKEVGNLYDMFHTRNSLHRRAYQHK

                            430       440       450       460       470       480
mSAMHD1_isoform1    ISNLIDIMITDAFLKADPYVEITGTAGKKFRISTAIDDMEAFTKLTDNIFLEVLHSTDPQ
mSAMHD1_isoform2    ISNLIDIMITDAFLKADPYVEITGTAGKKFRISTAIDDMEAFTKLTDNIFLEVLHSTDPQ
hSAMHD1             VGNIIDTMITDAFLKADDYIEITGAGGKKYRISTAIDDMEAYTKLTDNIFLEILYSTDPK

                            490       500        510       520       530
mSAMHD1_isoform1    LSEAQSILRNIECRNLYKYLGETQPKR.EKIRKEEYERLPQEVAKAKPEKAPDVELKAED
mSAMHD1_isoform2    LSEAQSILRNIECRNLYKYLGETQPKR.EKIRKEEYERLPQEVAKAKPEKAPDVELKAED
hSAMHD1             LKDAREILKQIEYRNLFKYVGETQPTGQIKIKREDYESLPKEVASAKPKVLLDVKLKAED

                 540       550       560       570       580       590
mSAMHD1_isoform1    FIVDVINVDYGMEDKNPIDRVHFYCKSNSKQAVRINKEQVSQLLPEKFAEQLIRVYCKKK
mSAMHD1_isoform2    FIVDVINVDYGMEDKNPIDRVHFYCKSNSKQAVRINKEQVSQLLPEKFAEQLIRVYCKKK
hSAMHD1             FIVDVINMDYGMQEKNPIDHVSFYCKTAPNRAIRITKNQVSQLLPEKFAEQLIRVYCKKV

                 600       610       620       630       640       650
mSAMHD1_isoform1    DGKSLDAAGKHFVQWCALRDFTKPQDGDIIAPLITPLKWNNKTSSCLQEVSKVKTCLKF.
mSAMHD1_isoform2    DGKSLDAAGKHFVQWCALRDFTKPQQCGAGEMAEDPDSIPSTQQP.....HAAHNQL...
hSAMHD1             DRKSLYAARQYFVQWCADRNFTKPQDGDVIAPLITPQKKEWNDSTSVQNPTRLREASKSR

mSAMHD1_isoform1    .........
mSAMHD1_isoform2    .........
hSAMHD1             VQLFKDDPM
```

Supplementary Figure 3. Human and mouse SAMHD1 proteins are similar in sequence. (**a**) Schematic of SAMHD1 proteins and mutants. (**b**) Sequence alignment of hSAMHD1, mSAMHD1 iso1, and mSAMHD1 iso2. Created with ESPript (http://espript.ibcp.fr).

Supplementary Figure 4

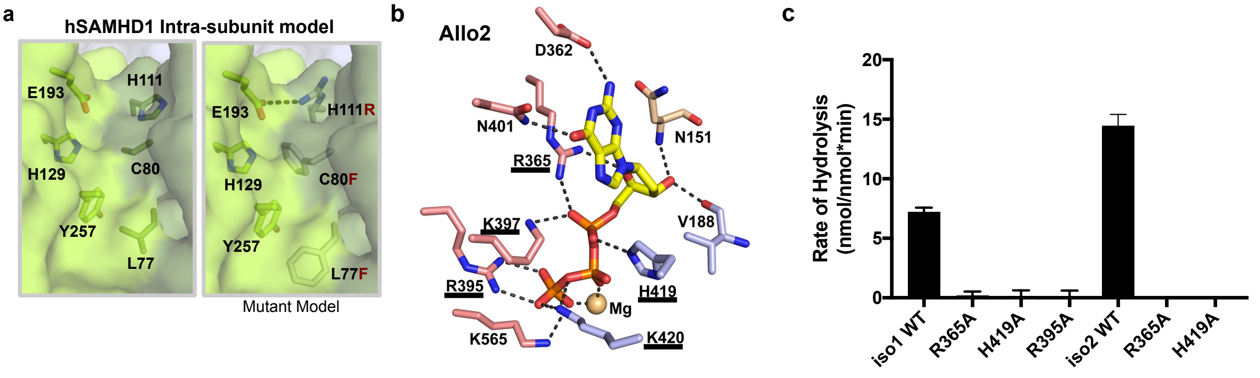

Supplementary Figure 4. Nucleotides binding at allosteric site 2 is important for mSAMHD1 activity. (**a**) Left, model of hSAMHD1 SAM-to-HD intra-subunit interface based on the 2-Allo mSAMHD1 structure. Right, model of SAM-to-HD interface after hSAMHD1 residues are mutated to the corresponding mSAMHD1 residues. (**b**) Residues interacting with the dNTP at Allo-site 2. (**c**) dNTPase activity assay for WT and mutants of mSAMHD1 iso1. Reaction products were quantified by the malachite green assay. Each experiment was performed in triplicate. Error bars, s.d.

Supplementary Figure 5

a

Between four independent subunits in the No-Allo structure

	A	B	C	D
A		0.49	0.56	0.77
B			0.52	0.79
C				0.55
D				

b

Between two independent subunits in the 1-Allo structure

	A	B
A		0.85
B		

c

Between subunits (chain A) from No-Allo, 1-Allo, and 2-Allo structures

	No-Allo	1-Allo	2-Allo
No-Allo		1.12	1.11
1-Allo			0.31
2-Allo			

d

Between the two independent rigid dimers in the No-Allo structure

	A	B
A		0.54
B		

e

Between the independent rigid dimers from No-Allo, 1-Allo, and 2-Allo structures

	No-Allo	1-Allo	2-Allo
No-Allo		1.06	1.33
1-Allo			0.7
2-Allo			

f

Between tetramers from No-Allo, 1-Allo, and 2-Allo structures

	No-Allo	1-Allo	2-Allo
No-Allo		3.19	2.41
1-Allo			2.86
2-Allo			

Supplementary Figure 5. Structural comparisons of the components of mSAMHD1 tetramers in the three activation states. (**a**) RMSD values from pairwise alignments of the monomers within the No-Allo structure. (**b**) RMSD from alignment of the monomers within the 1-Allo structure. (**c**) RMSD values from pairwise alignments of the monomers between the No-Allo, 1-Allo, and 2-Allo structures. (**d**) RMSD from alignment of the two independent rigid dimers within the No-Allo structure. (**e**) RMSD values from pairwise alignments of mSAMHD1 rigid dimers between the No-Allo, 1-Allo, and 2-Allo structures. (**f**) RMSD values from pairwise alignments of mSAMHD1 tetramers between the No-Allo, 1-Allo, and 2-Allo structures.

Supplementary Figure 6

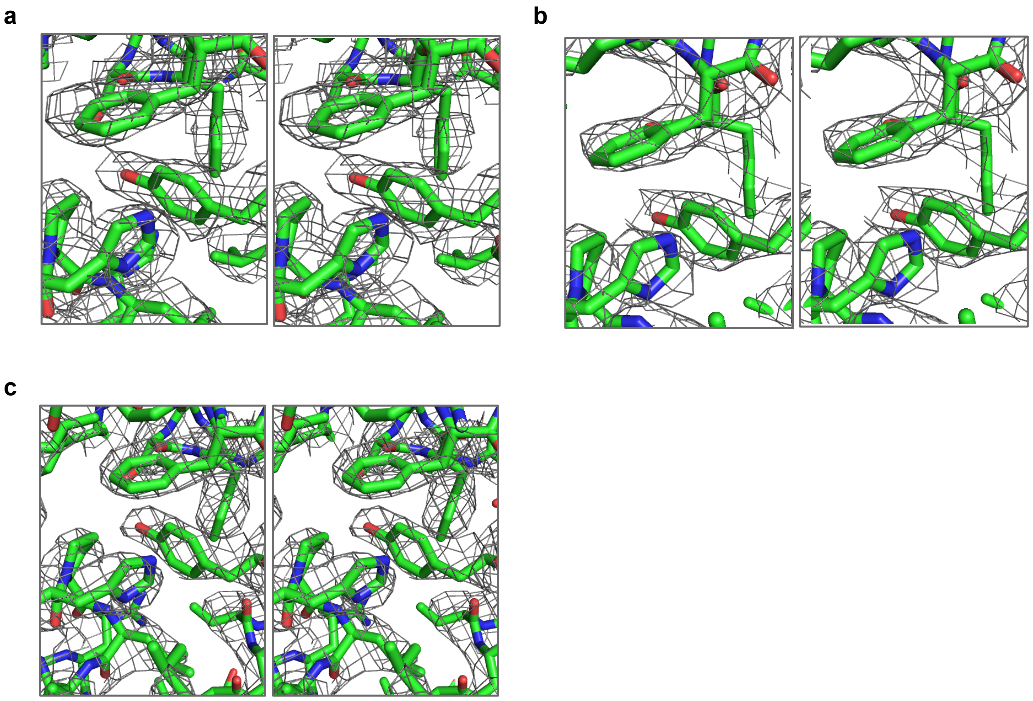

Supplementary Figure 6. Stereo images of electron density. Stereo images of a portion of (**a**) No-Allo crystal structure and (**b**) 1-Allo crystal structure and (**c**) 2-Allo crystal structure. The 2Fo-Fc electron density maps countoured at 1 σ shown as black mesh.

Supplementary Figure 7

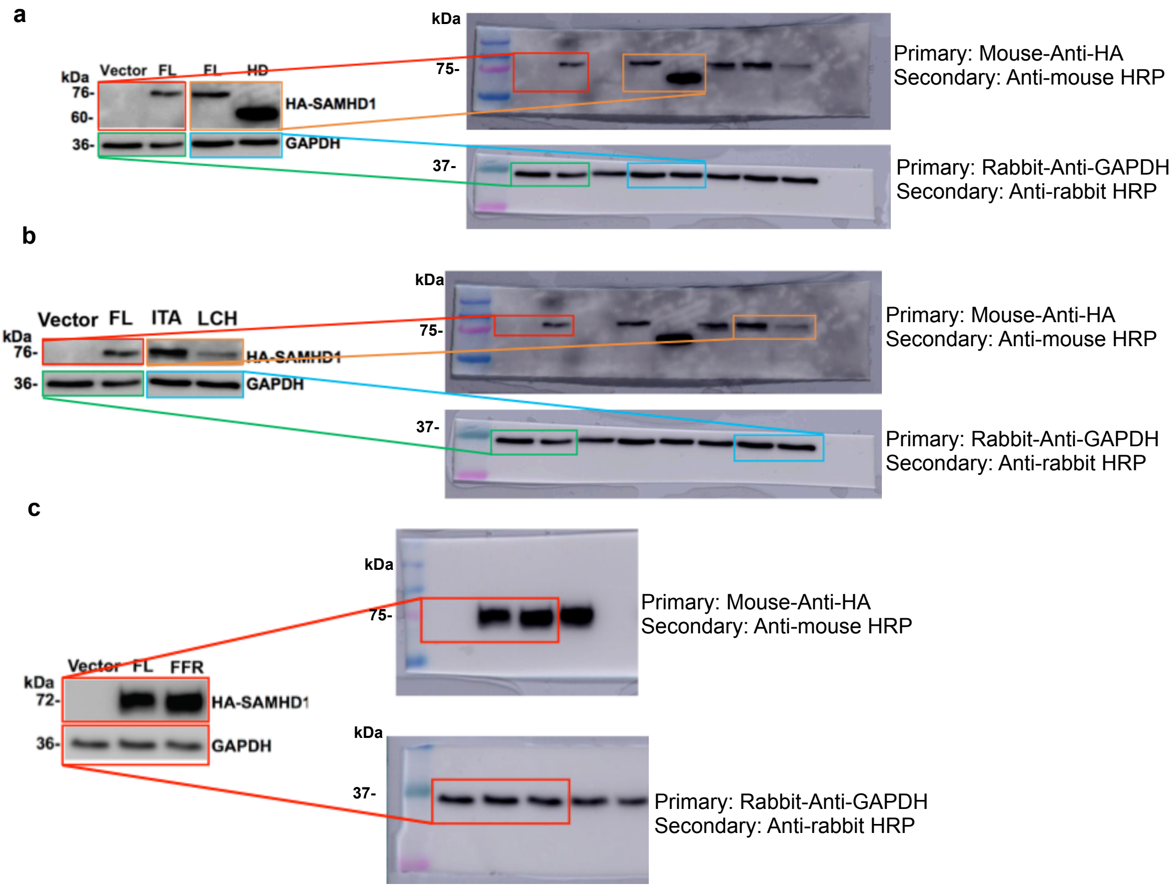

Supplementary Figure 7. Uncropped immunoblots. (**a**) Immunoblots corresponding to figure 1i. (**b**) Immunoblots corresponding to figure 3c. (**c**) Immunoblots corresponding to figure 3f.

Supplementary Table 1: Primers used in the study

Clone	Primers
hSAMHD1 FL	Forward: GT ggatcc atgcagcgagccgattccgagcagccc<br><br>Reverse: CTT ACC GGT TCA AGC ATA ATC TGG AAC ATC ATA TGG ATA CAT TGG GTC ATC TTT AAA AAG CTG GAC TCT GCT TTT GGA TGC
mSAMHD1 iso1 FL	Forward: GT ggatcc atggactcacttttggggtgtggtgtc<br><br>Reverse: CTT ACC GGT TCA AGC ATA ATC TGG AAC ATC ATA TGG ATA AAA TTT TAG ACA TGT TTT TAC TTT GGA TAC TTC TTG GAG GC
mSAMHD1 iso2 FL	Forward: GT ggatcc atggactcacttttggggtgtggtgtc<br><br>Reverse: CTT ACC GGT TCA AGC ATA ATC TGG AAC ATC ATA TGG ATA CAG CTG GTT GTG AGC CGC ATG TGG CTG CTG
mSAMHD1 iso1 HD	Forward: GT ggatcc atg GAT CTA ATG AAG GTA TTT AAT GAT CCC ATT CAT GGC CAC<br><br>Reverse: CTT ACC GGT TCA AGC ATA ATC TGG AAC ATC ATA TGG ATA AAA TTT TAG ACA TGT TTT TAC TTT GGA TAC TTC TTG GAG GC
mSAMHD1 iso2 HD	Forward: GT ggatcc atg GAT CTA ATG AAG GTA TTT AAT GAT CCC ATT CAT GGC CAC<br><br>Reverse: CTT ACC GGT TCA AGC ATA ATC TGG AAC ATC ATA TGG ATA CAG CTG GTT GTG AGC CGC ATG TGG CTG CTG
hSAMHD1 HD	Forward: GT GAATTCacaatgaaggt aattaatgat<br><br>Reverse: CTT ACC GGT TCA AGC ATA ATC TGG AAC ATC ATA TGG ATA CAT TGG GTC ATC TTT AAA AAG CTG GAC TCT GCT TTT GGA TGC
mSAMHD1 RN	Forward:<br>ctgtgttcagattgcggggctctgccggaacctaggtcatgggccattttctcatatg<br>Reverse:<br>catatgagaaaatggcccatgacctaggttccggcagagccccgcaatctgaacacag
hSAMHD1 RN	Forward:<br>**gttcagattgctggactttgt**CGTAAT**ctcggtcatgggccattttc**<br>Reverse: gaaaatggcccatgaccgagattacgacaaagtccagcaatctgaac

Supplementary Table 1 continued

mSAMHD1 ITA mutant	S142I Forward: GAA TGT ATC CAG CAG CTG AGT CAG att CGG ATT GAT CTA ATG AAG GTA TTT AAT GAT CCC<br>S142I Reverse: GGG ATC ATT AAA TAC CTT CAT TAG ATC AAT CCG AAT CTG ACT CAG CTG CTG GAT ACA TTC<br>SN566-567TA Forward: CGA TCG TGT TCA CTT CTA TTG TAA G acc gcc AGC AAG CAA GCG GTC AGG ATC<br>SN566-567TA Reverse: GAT CCT GAC CGC TTG CTT GCT GGC GGT CTT ACA ATA GAA GTG AAC ACG ATC G
hSAMHD1 LCH to FFR mutant	L77F,C80F Forward: CCG AGA AAA TGA AAT CAC AGG CGC A TTT ctgcct TTT cttgatgagtctcgttttgaaaatcttg<br>L77F,C80F Reverse: CAA GAT TTT CAA AAC GAG ACT CAT CAA GAA AAG GCA GAA ATG CGC CTG TGA TTT CAT TTT CTC GG<br><br>H111R Forward: CTT AGT TAT ATC CAG CGA TTG GTT CAA ATC cgc GTT GAT ACA ATG AAG GTA ATT AAT GAT CCT ATC<br>H111R Reverse: GAT AGG ATC ATT AAT TAC CTT CAT TGT ATC AAC GCG GAT TTG AAC CAA TCG CTG GAT ATA ACT AAG
mSAMHD1 FFR to LCH mutant	FF109,112LC Forward: gagacaataaaatcgccggctcg CTG ctgccc tgt TTG GAT GAG GAT CGT CTG GAA GAT CTG<br>FF109,112LC Reverse: AG ATC TTC CAG ACG ATC CTC ATC CAA ACA GGG CAG CAG CGA GCC GGC GAT TTT ATT GTC TC<br>R143H Forward: GTA TCC AGC AGC TGA GTC AGT CT cac attgatctaatgaaggtatttaatgatccc<br>R143H Reverse: GGG ATC ATT AAA TAC CTT CAT TAG ATC AAT GTG AGA CTG ACT CAG CTG CTG GAT AC
mSAMHD1 R365A mutant	R365A Forward: CTT GGA ATC CAA AAT AAT TTT GAT TAC AAG gcc TTC ATT AAG TTT GCC CGT ATC TGT G<br>R365A Reverse: CAC AGA TAC GGG CAA ACT TAA TGA AGG CCT TGT AAT CAA AAT TAT TTT GGA TTC CAA G
mSAMHD1 H419A H420A mutant	H419K420AForward: CTT ACA CCG AAG AGC TTA CCA A gccgcg ATC AGC AAC CTC ATC GAC ATA

	ATG<br>H419K420A Reverse: CAT TAT GTC GAT GAG GTT GCT GAT CGC GGC TTG GTA AGC TCT TCG GTG TAA G
mSAMHD1 R395A mutant	R395K397A Forward: GTA AGG TGA AGC ACA TTT GTT CG gcagaagcg GAG GTT GGA AAT CTG TAT GAC ATG<br><br>R395K397A Reverse: CAT GTC ATA CAG ATT TCC AAC CTC CGC TTC TGC CGA ACA AAT GTG CTT CAC CTT AC
