# Supplementary material for: The SAM domain of mouse SAMHD1 is critical for its activation and regulation
Source: Nat Commun. 2018 Jan 29;9:411. doi: 10.1038/s41467-017-02783-8 (PMC5788916; doi:10.1038/s41467-017-02783-8)
Supplement: Supplementary file 3 — Description of Additional Supplementary Files [file 41467_2017_2783_MOESM3_ESM.pdf]

Description of Supplementary Files

File Name: Supplementary Movie 1
Description: A view of the swinging motions between the two rigid mSAMHD1 dimers across the No-Allo, 1-Allo, and 2-Allo tetramer states. The animation is created from the three mSAMHD1 crystal structures.

File Name: Supplementary Movie 2
Description: The animation in Supplementary Movie 1 rotated 90°. The movie shows the changes in compactness between the No-Allo, 1-Allo, and 2-Allo tetramer states. The animation is created from the three mSAMHD1 crystal structures.
