# Supplementary figures and images for: The SAM domain of mouse SAMHD1 is critical for its activation and regulation
Source: Nat Commun. 2018 Jan 29;9:411. doi: 10.1038/s41467-017-02783-8 (PMC5788916; doi:10.1038/s41467-017-02783-8)

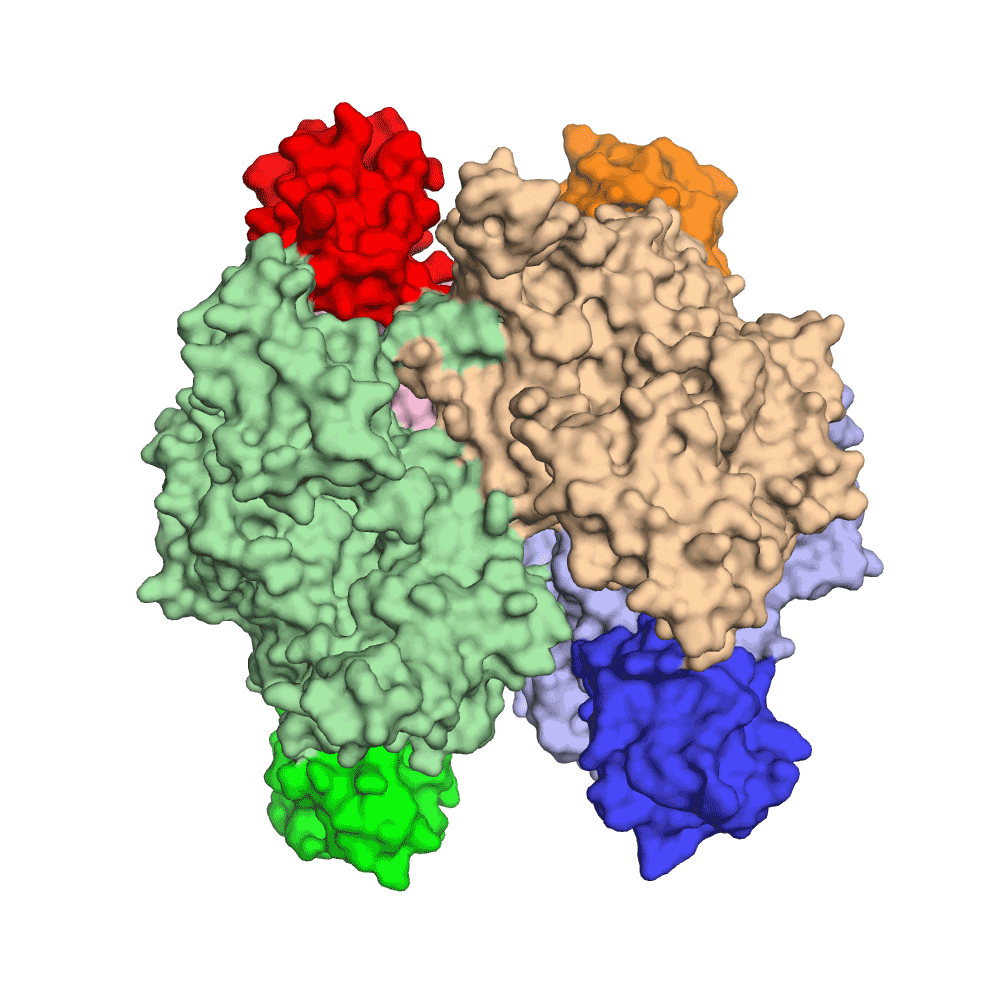

Supplement: Supplementary file 4 — Supplementary Movie 1 [file 41467_2017_2783_MOESM4_ESM.gif]

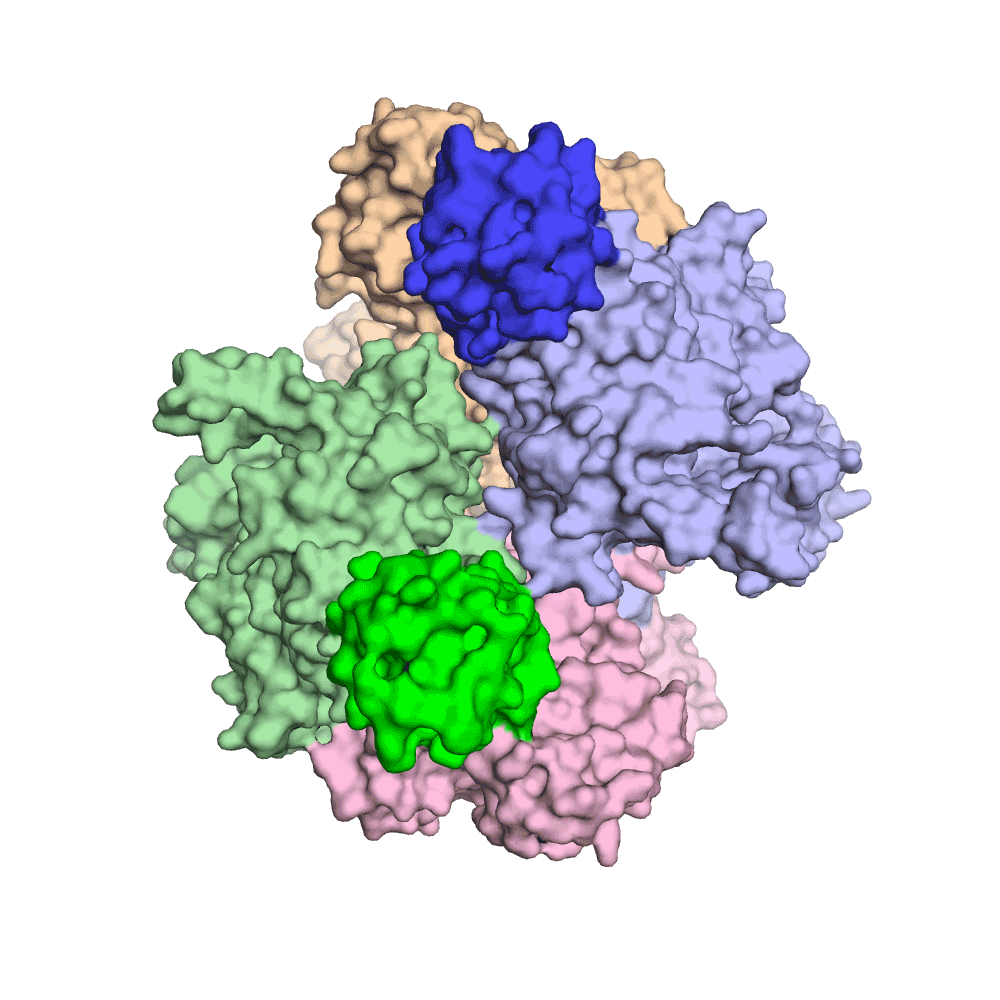

Supplement: Supplementary file 5 — Supplementary Movie 2 [file 41467_2017_2783_MOESM5_ESM.gif]
